# VHH selected against the viral spike protein can protect mice against lethal rabies virus challenge

**Published:** 2011-01-10

**Authors:** Valérie Rosseels, Peter Vanlandschoot, Anna Hultberg, Theo Verrips, Michael Saunders, Hans de Haard, Aurélie Francart, Michael M Kalai, Steven Van Gucht

**Affiliations:** 1 Rabies Laboratory, Communicable and Infectious Diseases, Scientific Institute of Public Health, Brussels, 1180, Belgium; 2 Ablynx NV, Gent, 9052, Belgium; 3 Cellular Architecture & Dynamics, Department of Biology, University of Utrecht, Utrecht, 3500 The Netherlands

## 

VHH are polypeptides (15 kDa) derived from the variable domain of single heavy-chain antibodies of *Camelidae*. They represent the smallest antigen-binding fragment of an antibody (human IgG = 150 kDA). VHH are currently being explored for a number of applications.

Anti-rabies virus VHH were cloned from lymphocytes of vaccinated llamas and selected for their affinity with the viral spike glycoprotein G and neutralizing potency in a cellular infection assay. Linkage of two VHH allowed recognition of two identical or different epitopes and increased the neutralizing potency in cells more than a hundred-fold. Next, we examined the protection efficiency of these bimeric VHH *in vivo*.

Contrary to irrelevant control VHH, pre-incubation of the virus with anti-G VHH fully protected mice against disease and mortality upon inoculation of the virus-VHH mix in the nose, muscle or brain. Preventive administration of anti-G VHH in the nose, 24 hours prior to intranasal virus challenge, also almost completely prevented disease and lethal infection. This suggests that VHH remain sufficiently active for at least 24 hours at the site of administration in the nose to neutralize a significant part of the invading virus. Post exposure prophylaxis (PEP) by injection of anti-G VHH in the left quadriceps muscle 10 minutes after virus challenge in the right quadriceps muscle reduced mortality by 50%. Treatment 24 hours after virus challenge was however no longer effective, most likely because the virus had already reached the central nervous system and was no longer exposed to locally administered VHH.

Our results show that anti-G VHH can neutralize rabies virus in an Fc-independent way. VHH probably hinder the recognition of cellular receptors or interfere with the fusion of viral and cellular membranes. The bimeric constructs proved protective in different challenge models, but that protection in the PEP model was weak. This might be due to the short half-life of the used VHH. Considering that prolongation of the half-life of VHH is feasible by different approaches, VHH technology may offer perspectives as an alternative to antibodies for PEP. VHH have a low production cost, not contaminated by blood-borne pathogenic agents and are less likely to evoke allergic or immunopathological reactions. They have good thermal stability, which is an advantage in developing countries, where the cold chain for distribution and preservation can not always be guaranteed.

